# Centering Biodiversity: Integrating the Complexity of Sex Into a Developmental Biology Course

**DOI:** 10.1093/icb/icag149

**Published:** 2026-08-10

**Authors:** A M Aramati Casper, Brandon Hylton, Deborah M Garrity

**Affiliations:** Biology Department, Colorado State University, Fort Collins, CO 80523, USA; Graduate Degree Program in Ecology, Colorado State University, Fort Collins, CO 80523, USA; Biology Department, Colorado State University, Fort Collins, CO 80523, USA; Biology Department, Colorado State University, Fort Collins, CO 80523, USA

## Abstract

In biology, variation is the norm, not the exception. This particularly holds true for the concepts of sex and sex determination, which we refer to here as *biology of sex*. Unfortunately, biology of sex topics are often taught using oversimplified content, situating sex as a simple, genetically determined binary, and that mammals are the predominant model for biology of sex across organisms. Teaching using these oversimplifications leads students to develop a limited understanding of biology of sex topics, which limits their capacity as future researchers and also harmfully and erroneously supports discriminatory social norms and legislation. To work towards developing effective pedagogical strategies that teach the complexity of biology of sex topics, we used the knowledge in pieces (KiP) theoretical framework to explore the question, *how did students’ knowledge of sex and sex determination change after a revised unit that centered biologically diverse examples and biological mechanisms* in an advanced undergraduate level developmental biology course. Twenty-five of the 48 enrolled students participated in our study. We analyzed students’ responses to a reflection and a pre-post assessment using reflexive thematic analysis and compared students’ exam responses to multiple-choice questions about biology of sex topics and other topics. Our qualitative and quantitative results demonstrated that the expanded unit had mixed effectiveness. From their reflections, students indicated that they thought learning about biology of sex topics was important as part of their biology education and identified the current cultural climate as a reason for discussing, not avoiding, these topics. However, students disproportionately struggled with learning this content. On the exam questions, students performed more poorly on the biology of sex topics than on the other topics covered on the same exam. Their pre-post assessments revealed that student responses changed to responses aligned with the course content more easily for subtopics that were more likely to be novel than for more familiar and often socially charged subtopics. These results demonstrate that seemingly simple topics that are societally entrenched, such as defining the concept of sex, may be more difficult to shift than more conceptually complex concepts that are less entrenched.

## Introduction

Biology is complex and comprises almost unimaginable variation (Bachtrog et al. 2014; Gorelick et al. 2016; Leonard 2018). Yet, ironically, biology content is commonly oversimplified for teaching—even to the point of teaching incorrect information—in order to fit curricular needs. The topics of sex, defined here as an organismal characteristic, and sex determination, collectively termed “biology of sex” in this manuscript, offer a compelling example of a complex landscape of concepts, non-intuitive vocabulary with nuanced and overlapping meanings, and broad range of species-specific mechanisms, all of which must be reduced in scope and communicated as clearly as possible to students (Bazzul and Sykes 2011; Casper et al. 2022; Casper, Fuselier, et al. 2025). One challenge in teaching about sex-related topics is the term *sex* itself (Eppley et al. 2026; Massa 2026). This short word is used to describe a range of concepts, including the process of meiosis and syngamy, gamete compatibility related to size differences, the types of gametes an organism produces, or actions and behaviors that work to bring gametes in close proximity to facilitate syngamy (Warkentin et al. 2026). When referencing an organismal characteristic in anisogamous species (those that have two different types of gametes, in which size is relevant to compatibility), sex can refer to the type or types of gametes an individual makes and the many characteristics associated with a specific type of gamete production (e.g., hormones, gonads, chromosomes; Warkentin et al. 2026). Indeed, in this issue of Integrative and Comparative Biology, Warkentin et al. (2026) describe seven biologically distinct concepts or processes associated with the word *sex*. In response to such multivalent, ambiguous and overlapping meanings, some have proposed that the term *sex* be strictly defined by gamete type, and limited to this context alone (Goymann et al. 2023). Conversely, others propose that sex should be understood to encompass a multivariate suite of traits, which researchers should parse out into separable factors, each to be individually measured and statistically compared, rather than collapsing a cohort of diverse biological data under a single label (e.g., Maney et al. 2020; Anderson and Falk 2023; McLaughlin et al. 2023; McDonough-Goldstein et al. 2026). As part of this discussion, there are growing calls for researchers to define the term sex in their work, with some journals now requiring authors to define sex in their papers (Cell Press 2026; Eppley et al. 2026).

While these conceptual discussions in biology are important, revisions to the content we present when teaching about the biology of sex cannot be indefinitely delayed until a more coherent framework is developed. Teaching content that includes the variation and complexity of biology of sex topics is important to support authentic descriptions of biology, and the authentic diversity of humans in both biology classrooms and within society (Casper et al. 2022; Lewis and Sharpe 2023; Casper, Fuselier, et al. 2025; Eddy et al. 2026a). A critical first step is to select content that centers biological diversity when teaching biology of sex topics (Long et al. 2021; Štrkalj and Pather 2021; Zemenick et al. 2022, 2023; Harris et al. 2024; Casper, Fuselier, et al. 2025; Casper 2026). However, there is a lack of classroom-based research regarding whether simply integrating complexity into existing pedagogical strategies is effective for deepening students’ perceptions on the topic.

Teaching biology of sex has another specific challenge: students enter the classroom with ingrained prior knowledge. Much of this knowledge is acquired at a young age and is frequently highly mammal-centric and often partially at odds with scientific findings. Research supports the contention that the information people initially learn has robust staying power, whereas subsequent new, conflicting information is more difficult to learn (Posner et al. 1982; Scantlebury and Martin 2010; Hadjiachilleos et al. 2013; Heddy and Sinatra 2013; Vidak et al. 2019; Harlow and Bianchini 2020). Hence, the strategy of teaching students oversimplified content may make it harder for them to embrace complexity later. Conversely, teaching intricate, nuanced content can be more challenging for instructors, as textbooks frequently portray an oversimplified, binary-centric perspective (Bazzul and Sykes 2011; Fuselier et al. 2018; Ray King et al. 2021; Spaulding and Fuselier 2023; Donovan et al. 2024; Jackson et al. 2024).

Understanding complexity about sex-related topics in biology has significant ramifications beyond the classroom. Currently, biology is being used erroneously to support laws and policies rooted upon inaccurate, discriminatory information (e.g., The Florida Legislature 2026; Exec. Order No. 13,950 2020). Students are aware that this content has societal relevance, and students recruited across the U.S. for a study about how biology of sex topics were taught felt that it is important that those earning biology degrees should be able to articulate scientifically supported viewpoints biology of sex topics (Casper et al. 2022; Eddy et al. 2026a). Thus, teaching breadth and complexity of the biology of sex is important for multiple reasons.

The inherent complexity of sex-related topics, coupled with the differences between societal dialogues and scientific dialogues about biology of sex, supports the need for research for effective instruction of these topics. Our research differs from previous research about these topics (e.g., Casper et al. 2022; Eddy et al. 2026a) because we studied the experiences of all of the students in one course, rather than only one or a few students in a range of courses across the U.S. In this manuscript we use the education theory of knowledge in pieces (described below) to explore how content that centered biological diversity about sex determination influenced student learning about biology of sex in an advanced undergraduate developmental biology course, and whether students were receptive to a larger module dedicated to this content.

### Theoretical framework

We used the theoretical framework of knowledge in pieces (KiP) as an analytic lens in our study. As such, we used it to interpret how students were drawing from material presented in the course and other sources of information. The KiP theoretical framework provides insights into the ways students access, revise, and reorganize information, gradually developing increased coherence in their understanding as they learn (Harlow and Bianchini 2020; Lira 2020). Specifically, KiP is a constructivist learning theory that conceptualizes naïve understanding as starting with small, loosely integrated knowledge elements that an individual draws upon, often in a context-dependent fashion (Harlow and Bianchini 2020). These pieces are themselves neither inherently right or wrong, but instead reflect intuitive experience-based conceptions activated as one grapples with new knowledge (Lira 2020). Learners gradually evolve and refine their knowledge elements, or “knowledge pieces” to achieve more coherent, sophisticated explanations, using an iterative and often individualized process (Lira 2020). Counter examples may challenge some or all of these elements, causing students to realize their initial knowledge does not apply universally (Lira 2020). In the academic classroom, the instructor gradually introduces explanations grounded in different disciplines (e.g., genetics, anatomy, hormones, and environmental factors), thus challenging students to expand their initial loosely structured knowledge pieces into more complex versions that account for the context (e.g., the species, the developmental stage, and the life history pattern). Hence, students begin to move from monofactorial to contextually dependent multifactorial explanations (Lira 2020). KiP suggests that as the students engage with the learning activities, they start to integrate the disparate pieces of new information alongside their prior, culturally informed knowledge to achieve a more nuanced understanding (Harlow and Bianchini 2020). Thus, rather than replacing their initial ideas wholesale, they reframe them to produce more flexible and scientifically accurate explanations (Harlow and Bianchini 2020; Lira 2020).

The principles of KiP provide guidance in supporting student learning in a context where a seemingly simple term, sex, has a multiplicity of meanings and common implicit assumptions; thus, navigating this complexity involves a plethora of distinct but inter-related concepts (Sanz 2017; McLaughlin et al. 2023; Casper et al. 2026; Massa 2026; McDonough-Goldstein et al. 2026; Warkentin et al. 2026). Table 1 provides several examples of knowledge pieces connected to biology of sex concepts that students commonly hold when they enter the course. As the course progresses, more complex explanations unfold that challenge students to expand, re-frame, or qualify their initial ideas depending on the context, requiring them to evaluate a range of potentially conflicting knowledge pieces and select those that are relevant in a given situation.

**Table 1:** Example biology of sex topics, common prior knowledge, and their biological complexity

Topic	Prior knowledge	Biological complexity
The process of sex determination	Chromosomes determine sex.	Sex determination is a **complex multi-component process** that involves a range of factors across different species, including hormones, gene regulation, and environmental factors.
Phenotype of XX and XY individuals	XX individuals are phenotypically female and XY individuals are phenotypically male	Even in humans, **multiple components interact non-linearly** in development to create a bimodal (not binary) distribution of phenotypes. Characteristics often assumed to be aligned—including chromosomes, hormones, gonads, and genitals—can vary independently. For example, someone with XY chromosomes and testes might develop female-typical external anatomy due to androgen insensitivity. **Systems-level reasoning** is required to make sense of the interaction between genetics, hormones, anatomy, puberty, and fertility.
Plasticity of sex-related traits in an individual	Sex-related traits are fixed.	Sex-related traits can **change over time**; they are **emergent and dynamic**.
Nature of sex categories	Sex categories exist as a fixed binary of male and female.	Interpreting the vast variation in sex-related traits as a set of categories, such as male, female, intersex, or hermaphrodite invokes a **societally constructed context**. Navigating the social construction of sex requires **critical thinking** about how **biology intersects with culture and identity**.

### Research questions

We used the lens of KiP to analyze how students’ explanations of biology of sex topics developed in an upper-level undergraduate developmental biology course that centered the biological diversity related to biology of sex topics. KiP was particularly useful in our situation because it helped us to understand how students situated the knowledge pieces from the course in larger societal dialogues about sex-related topics and analyze the different knowledge pieces that students were drawing from on their course assessment. Specifically, we studied:

1. How did students’ knowledge of sex and sex determination change after an expanded unit that centered biologically diverse examples and biological mechanisms?
2. Did students feel that learning about biology of sex in depth is important and relevant for students majoring in biology or similar majors?

## Methods

### Course context

This study was performed in an upper-level developmental biology course during the 8-week summer 2023 session at a research-intensive university in the western U.S. The course was taught by the last author (DG), a biology professor with 20 years of teaching experience. As an upper-level undergraduate course, students were primarily college-level juniors and seniors. Prior coursework included a prerequisite introductory biology course that presented core aspects of biology of sex in animals and plants from a comparative, organism-focused perspective. Additional coursework in molecular genetics presented sex from the perspective of how genes work, covering chromosomal sex determination, meiosis, gamete formation, sex-linked inheritance, population genetics relevant to sexual reproduction, and gene regulation in sexual development. Neither course deeply emphasized the hormonal regulation of sex, detailed molecular mechanisms of sex determination, or social, cultural, or identity-based aspects of sex. There were 48 students in the course and in-person attendance hovered around 50%, although formal attendance was not taken in lecture. Lecture recordings were available on the course management system. The lecture met 3 days a week for two consecutive 50-minute sessions (100 minutes). The course also had a required lab, which is not studied in this paper. The labs involved active engagement with experimentation and met once a week for 3 hours, 20 minutes. The lecture used a blended instruction approach with short lectures integrated with active learning activities including in-class worksheets, brief guided discussions, think-pair-share, clicker questions, in-class problem-solving/predicting outcomes and an out-of-class reflection (delivered as an online survey). As our focus in this study was on integrating more nuance of content about biology of sex, and not studying content delivery strategies, the same types of content delivery strategies were used for this module as throughout the course.

### Intervention

In the semester of this study, the developmental biology curriculum was expanded from our typical two 50-minute class periods on biology of sex to five 50-minute class periods. This change allowed deeper engagement with definitions and concepts, featured biologically diverse examples, and emphasized mechanistic explanations. These modules presented revised or newly developed content to teach the concepts of sex and sex determination, with topics listed in Supplement 1. All authors collaboratively developed content that centered the biological diversity of sex and sex determination, and the evolution of sex determination systems. Materials were taught by the instructor and GTA. During the material development phase and teaching, we aimed to integrate this content into the course structure rather than providing the content as a series of “fun facts,” as recommended by Eddy et al. (2026a).

### Recruitment and data collection

To recruit students for the study, the first author (AC) gave a short presentation about the research and distributed consent forms to students in the first week of class. AC was not involved in teaching the course nor grading, and neither the instructor (DG) nor GTA (BH) were in the room while students completed the consent forms. Twenty-five of the 48 students enrolled in the course consented to participate. All data were collected from assignments and activities that all students completed as part of the class. For this study we analyzed the pre- and post-assessments, the multiple-choice questions on one exam (exam three), and one reflection assignment. The identical in-class pre- and post-assessments were comprised of 11 open-ended questions developed collaboratively by the researchers (all relevant questions provided in text). The 8-week summer course consisted of 35 lecture periods (50 minutes each) interspersed by four exams. Students completed the pre-assessment in week 4, just prior to the biology of sex unit (lectures 18–22; weeks 4 and 5) and the post-assessment following exam 4 on the final day of class (end of week 8), about three weeks after the final biology of sex lecture. These ungraded assessments were completed in class and with only AC present for the pre-assessment and BH for the post-assessment (AC was unavailable) to help minimize any student concerns that their responses would influence their grade or the instructor’s perceptions of their learning. Exam 3 (start of week 7) included 50% biology of sex content and 50% content on the development of the central nervous system, eye, hair, muscle, and bone (lectures 23–27). It consisted of multiple-choice questions, short answer questions, and diagrams. Due to their relevance to our research questions, we only analyzed responses to multiple-choice questions. Prior to the exam students had access to a practice exam. In addition, some questions for both the biology of sex content and the other content on exam three were new and some were reused from prior exams. However, even for reused questions, students are not likely to have seen the exact questions asked since all reused questions were drawn from the instructor’s private test bank of 20 years’ worth of example questions.

Students completed the online reflection assignment in the final week of class. Fourteen consenting students completed the pre-assessment, 19 completed the post-assessment, and 11 completed both. Twenty-five consenting students completed exam three and 17 completed the reflection.

### Researchers

We each brought different perspectives to our collaborative research. AC is a research scientist in biology and education research. Ze helped the course instructor (DG) and graduate teaching assistant (GTA; BH) develop course materials. AC also guided course assessments, performed the initial analyses of the pre- and post-assessments, statistical analyses, and mentored the other authors in all data analyses. Ze had more than eight years of biology teaching experience and over 10 years of experience performing and leading education research at the time of this study. DG is a developmental biologist with over 20 years of teaching experience who has taught this class approximately 30 times. Her formal experience with education research is emerging. BH is a graduate student in biology studying developmental biology, with six years of lab teaching experience. He is new to education research. Collectively, researcher identities include white, African American, heterosexual, lesbian, cisgender, and non-binary. Thus, the research team includes three researchers with a range of identities and backgrounds that allow each to bring unique and valuable perspectives to the work and that intersect with the perspectives and cultural norms each researcher brings to biology of sex topics.

### Data analysis

We used a mixed-methods approach for analysis. We used reflexive thematic analysis (RTA; Braun and Clarke 2022) for our qualitative data, Wilcoxon signed-rank tests for our quantitative responses, and a concurrent triangulation design to integrate across our data types (Warfa 2016). This research was designated as exempt by the university Institutional Review Board #4464, which reviews all research that involves human subjects. An exempt designation indicates that the project poses no or minimal risk to participants and meets all other exempt designation criteria (National Institute of Health 2024).

We performed a deductive/inductive, semantic, experiential RTA, which was particularly suitable for our analysis because it explicitly engaged with the experiences of both the participants and the researchers. This approach allowed us to analyze the students’ knowledge as embedded in their socially constructed reality and draw from our own knowledge and experiences. AC coordinated the overarching data analyses and performed the primary coding of the pre-post assessments. DG was the primary coder of the exams and reflections. We used deductive coding for the pre-post data because each question had biologically accurate answers and inductive coding to characterize the range of ways students responded and characterize changes between the pre- and post-assessments. The coding was semantic because it engaged directly with the answers students gave and latent because it characterized changes in students’ understanding, going beyond characteristics explicit in the data. To analyze the reflection assignment, we used inductive coding that was both semantic and latent to characterize the explicit statements that students made and to consider the socio-cultural context prompting the responses. For both types of data, the coding was experiential because it engaged directly with students’ experiences in a course. As is conventional in RTA, the data were initially coded by a single researcher (Braun and Clarke 2022). Each researcher who led the analysis for a given type of data followed the six phases of RTA: (1) data familiarization, (2) systematic data coding, (3) generating initial themes, (4) developing and reviewing themes, (5) refining themes, and (6) writing the report (Braun and Clarke 2022). For phase one, the researcher leading the analysis read through the student responses. For the systematic data coding, the same researcher coded all responses, with a focus on how each student’s responses related to each other. To generate initial themes, the researcher iteratively read through the responses and previously taken notes. Then, the entire research team engaged in an iterative process of discussing, reviewing, and refining the themes for each dataset, including discussing theme development across types of data, following Braun and Clarke’s (2021) guidance for quality data analysis in RTA. We then collaboratively prepared our manuscript for publication.

To analyze patterns in student responses to questions for exam 3, BH partitioned the questions into the categories of “sex-related” or “other topic,” because we were interested in understanding if students were able to answer the biology of sex questions correctly at a similar rate to the other topics on the exam. We calculated the proportion of the questions that each student got correct for each type of question, creating a paired set of data. We performed the Shapiro-Wilk normality test in R, version 4.6 (shapiro.test), and determined that our data were not normally distributed. Therefore, AC performed a Wilcoxon signed-rank test in R, version 4.6, using the Wilcoxon test in the rstatix package (Kassambara 2026).

To integrate our qualitative and quantitative data, we used a concurrent triangulation design, centering our qualitative research (Warfa 2016). Specifically, we simultaneously drew from both our quantitative and qualitative data in our interpretations, focusing on the qualitative data to provide deeper insights into student meaning-making as well as context and nuance.

## Results and discussion

As presented below, our analyses of exam answers, an open-ended pre-post assessment, and student reflections indicated that students struggled to integrate material included in the biology of sex module. Indeed, students scored more poorly on the biology of sex topics than on other topics on the same exam, indicating that overall they were finding it challenging to identify the relevant biology of sex knowledge pieces and apply them to the course assessment, when compared to other similar topics. Of particular interest, the pre-post responses demonstrated that students often struggled to integrate new biology of sex knowledge pieces with existing knowledge pieces that potentially conflicted. Conversely, they struggled less when integrating new biology of sex knowledge pieces to which they had little or no prior exposure. In combination, the big-picture information from the quantitative analyses of the exam answers and the finer-grained details from the pre-post assessment and student survey demonstrate the importance of multifaceted data collection in understanding the challenges of teaching the complexity of biology of sex topics. Even with their struggles with the material, the majority of students strongly indicated they thought it was important and relevant to teach in-depth about biology of sex topics in this developmental biology course designed for STEM majors. Overall, these findings indicate the need for future research that implements targeted pedagogical strategies to support students with this integration.

### Exam grades

The analysis of students’ exam responses provides an overarching perspective of how well students were able to learn and use the information presented in class through selecting the appropriate knowledge pieces to apply to answer the exam questions. We compared student performance on multiple-choice questions about biology of sex topics compared to a second set of equally challenging, but generally novel subject matter on the same exam, which covered central nervous system, eye, muscle, and bone development. Comparing these sets of materials, we note that both included new vocabulary, new mechanisms, and specific gene functions, but the biology of sex material included several nuanced, overlapping vocabulary terms. Secondly, some of the biology of sex material was ostensibly familiar to students, whereas the other material tended to be largely novel for most students.

**Fig. 1:**
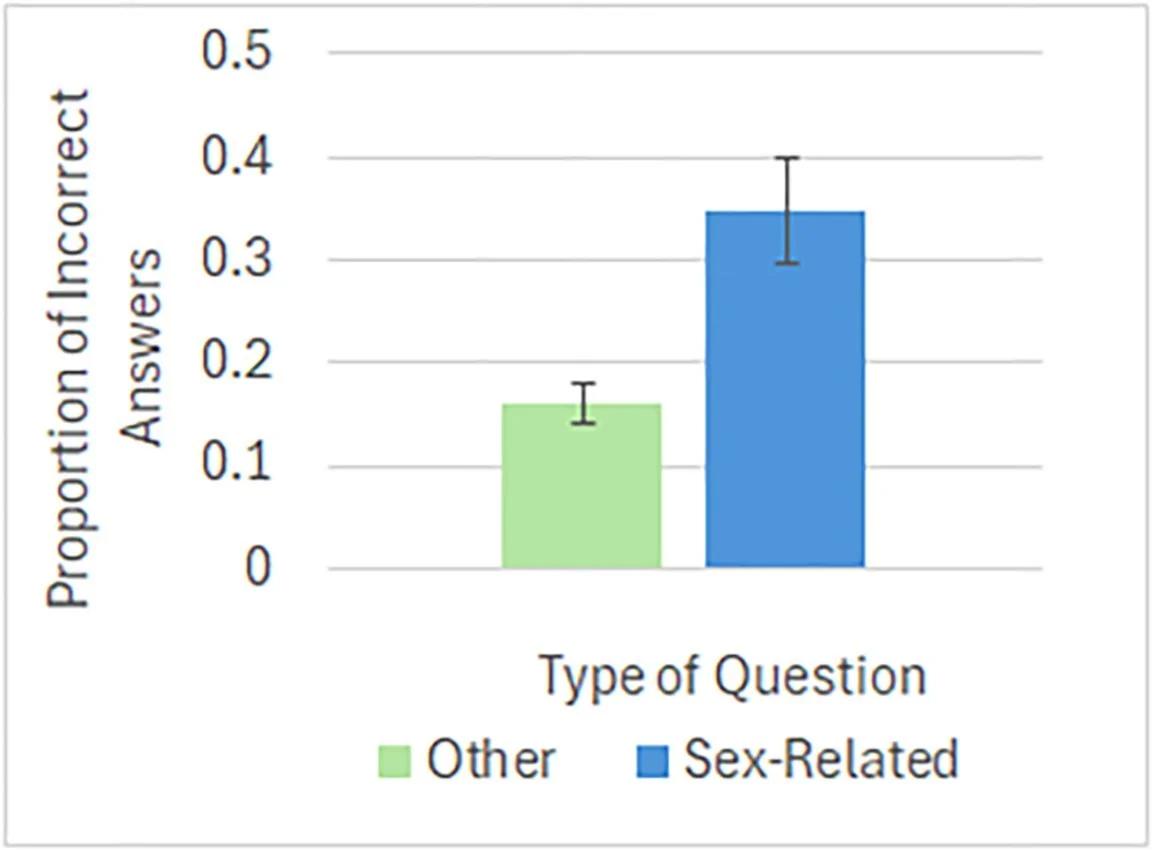
Proportion of incorrect answers for sex-related and all other questions on exam 3. Bars indicate standard errors.

We found that students performed more poorly on the biology of sex questions compared to the other topics (Fig. 1, Wilcoxon singed-rank test, Z = 3.6998, *p* = 0.0002). An average of 35% (*SD* = 12.9%) of students answered the biology of sex questions wrong, compared to an average of 16% (*SD* = 23.7) of students answering the questions on other topics incorrectly. The effect size is 0.805, which is large. These results demonstrate that students were less able to apply the relevant knowledge pieces from class for the biology of sex topics when compared to the other topics on the exam.

The incorrect answers that students chose indicated that they often struggled with nuanced definitions of specialized terms, especially those that have distinct yet related meanings. For example, students missed questions requiring them to evaluate the differences among hermaphrodite, gynandromorph, or intersex individuals. Another frequently missed example centered on the difference between sex determination and sexual systems. While the questions were written to evaluate students’ abilities to integrate salient knowledge pieces from the course to evaluate and analyze material presented, and not simply to test vocabulary knowledge, it is a frequent challenge of teaching biology that learners must grapple with a plethora of specialized technical terms, challenging them to both remember the unusual (and frequently long) new word, as well as match it to a distinctive meaning. Although vocabulary can be challenging broadly in biology, the vocabulary related to biology of sex can be particularly challenging because there are several long, probably new, words that all relate to what feels like a similar idea, such as hermaphrodite and intersex. In contrast, new vocabulary related to eye development (e.g., optic vesicle, morphogen) more clearly related to distinct concepts, so students needed only to assimilate the new word. Thus, it is possible that students’ language challenges influenced their performance on the assessment—if a student thinks a question is asking about sexual systems, and it is asking about sex determination, they will be less able to select relevant knowledge and apply it to the options provided. However, this is a real challenge in teaching about biology of sex topics, particularly since the language an individual has and uses influences their ability to develop conceptual understanding (Winawer et al. 2007; O’Connor 2019; Casper 2026; Warkentin et al. 2026).

Within the biology of sex questions students seemed to do better on questions about mammals than on flies (*Drosophila melanogaster*), although n-values were too small for meaningful statistical analyses. While both organisms operate with X and Y sex chromosomes, *Drosophila* sex determination mechanisms are very different than mammals. Here, complexity of mechanism rather than complexity of vocabulary appeared to be the primary challenge.

Overall, these exam results support the conclusion that vocabulary and concepts with overlapping but nuanced meanings, as well as non-mammalian mechanisms, were challenging obstacles for students as they navigated integrating the knowledge presented in the class into their pre-existing knowledge and then selecting the relevant knowledge pieces and applying them to the exam questions. While these data only provide an overarching picture, they demonstrate that students were less able to do this for the biology of sex material than for unrelated but equally complex developmental biology material.

### Pre-post assessment

Our analysis of the open-response pre-post assessment provides narrower but finer-grained insights beyond the exam responses about how students were integrating the course knowledge pieces into their pre-existing knowledge and collectively applying these knowledge pieces to the assessment. We developed two themes from the pre-post assessments that capture the changes in students’ responses between the pre- and post-assessments: (1) socially entangled concepts versus novel complexity and (2) sex is at least a little bit complex, even in humans.

#### Theme 1: Socially entangled topics versus novel complexity

Theme one centers the idea that the degree of success students demonstrated in integrating course knowledge pieces into their post-assessments often varied by how socially entangled the topic was, versus how novel the topic was. Students’ responses for topics more strongly associated with societally entrenched topics did not necessarily change to align with the course content easily. In contrast, more novel content sometimes elicited an “I don’t know” response in the pre-assessment, but later successfully generated a response grounded in course content for the post-assessment. We discuss this theme through two subthemes: (a) socially entangled concepts, and (b) novel complexity.

##### Subtheme 1a: Socially entangled concepts

Socially entangled concepts in the course included defining the term *sex*. The course presented five overlapping ways to classify or identify an individual’s sex in a given species. Of these, the type of gamete produced by the organism was emphasized as the most typical means for defining an individual’s sex. However, students’ answers to the question “*What are the different sexes that you are familiar with across all different types of organisms; b) Indicate the criteria for identifying each sex*” varied widely and did not necessarily align with course content by the time of the post-assessment.

Some students, such as #37, showed inconsistency in the way they addressed multiple questions related to defining sex on the pre-post assessments. For some questions, they effectively showed no change in their response, staying with oversimplified knowledge pieces and not referencing the knowledge pieces taught in the course. As one example, this student first wrote *“What an organism is born like, male or female (ex. XX or XY).”* In their post-assessment they wrote *“Biological, male/female.”* Thus, in this instance, student #37′s response demonstrated no engagement with more nuanced course content. In both assessments student #37 listed male, female, and hermaphrodite in response to *“What are the different sexes that you are familiar with across all different types of organisms*.” However, when asked if sex in humans is binary (Statement 1 from Theme 2, below), this student’s answer *did* change to engage with more complexity. Considering these answers collectively, this student entered the course with some knowledge pieces about how sex was more complex than just females and males, and further integrated more knowledge pieces about this complexity from the course. However, they did not always recognize that knowledge pieces related to this complexity were relevant to all prompts and did not generate consistent responses. Therefore, this student seems to be grappling with determining which knowledge pieces are relevant in a given context, rather than consistently answering from a perspective of integrated complexity.

For students whose responses changed between the pre- and post-assessments, the way they applied knowledge pieces in the post-assessment did not always align with the course. For example, student #19 described the different sexes they knew of in their pre-assessment as: *“Asexual—reproduces w/self, female—reproduces w/male, male—reproduces w/female.”* In their post-assessment they wrote *“Male: male chromosomes and testis, female: female chromosomes and ovary, intersex, simultaneous hermaphrodite, sequential hermaphrodite.”* Thus, this student changed their definition to integrate multiple types of relevant information yet did not include gametes in these lists, thus omitting a key criterion we emphasized as common for identifying an organism’s sex in both definitions. Their post-assessment definitions of male and female became less broadly applicable, although they no longer have an incorrect definition of asexual, and they added in intersex individuals and two types of hermaphrodites. Student #19′s post-assessment response is not specifically more aligned with common societal oversimplifications, but it also does not incorporate key material from the course.

Finally, some students did change the pieces of knowledge they were drawing from in ways that largely aligned with the course content in the post-assessment. For example, when describing the sexes they knew of and how these could be identified, student #67 wrote in their pre-assessment *“Females- often give birth, go through oogenesis, care for offspring (if applicable); Males- go through spermatogenesis, give less time to child rearing (if applicable); Hermaphrodite—is able to produce both female and male gametes, can reproduce asexually or sexually”* and in their post-assessment wrote: *“Female—large gametes, male—small gametes, intersex—ambiguous sex characteristics.”* Similarly, student #77 shifted from focusing on XX/XY genetics and animal-centric structures for sex in their pre-assessment: *“Male—XY, testes, penis, sperm; female—XX, ovaries, eggs, vagina; hermaphrodite—all structures as mentioned above (minus penis/vagina),”* to focusing on gamete production in their post-assessment *“Male—small gametes; female—large gametes; hermaphrodite—produces both large and small gametes, can self fertilize.”* Thus, they refined their response to focus on gamete production as stronger criteria for identifying an individual’s sex than behavior or anatomical structures, drawing from knowledge pieces taught in the course material. This change in knowledge pieces also aligned with broadly applicable characteristics, instead of focusing on characteristics that are limited to specific taxonomic groups.

Overall, our analysis of societally embedded biology of sex material identified inconsistent performance by students, with only some of them able to demonstrate course-aligned concepts by the post-assessment. Therefore, we observed a correlation with the societally charged subset of material, around which students have deeply entrenched but scientifically limited prior knowledge, and a reduced ability to consistently achieve learning gains that reflect the broader, more biodiverse and accurate concepts presented in the course.

##### Subtheme 1b: Novel complexity

We next considered topics that students were less likely to have encountered previously. The mechanisms of sex determination, including mechanisms that trigger sex changes in sequential hermaphrodites, were in this category. We found these topics more reliably aligned with the course content after instruction. We identified these less familiar topics through both our own experiences as instructors and because some students provided an “I don’t know” type of response for their pre-assessment responses. Two questions informed this theme: “*Once sex is determined during development in a vertebrate, is it fixed or can it change*” and *“Do all sexually reproducing organisms use the same sex determination mechanism as in vertebrates.”* For these questions, students who initially had answers not aligned with current biological knowledge on the pre-assessment typically provided course-aligned answers in the post-assessment. Conversely, no student who provided a course-aligned pre-assessment answer provided an un-aligned post-assessment answer.

For the prompt about sex determination in vertebrates, we observed variation in how successfully students’ responses integrated knowledge pieces from the course. For example, student # 37 initially wrote: *“I think in nature once sex is determined it’s not likely going to change,”* but wrote in the post-assessment, *“The individual’s sex could change, like in fish, some can change from female to male.”* Thus, student #37 reversed their opinion and integrated course knowledge. Student #40′s initial response drew from general knowledge about variation in biology, *“Not sure, but knowing biology there are probably ways to change it in both situations,”* but responded in the post-assessment with a specific example: *“In vertebrates sex can change in both scenarios, ”* moving from intuition to confidence in their knowledge. Student #65 added nuance and complexity to their post-assessment answer, changing from: *“I believe certain mutations can cause variability, ex: SRY mutation, I don’t believe it can be changed, just “fixed” to make life easier for the individual,”* to *“It can change based on environmental factors such as pH, temp, abundancy of opposite mate, etc*.”

Students’ responses about whether non-vertebrate organisms use the same sex determination mechanisms as vertebrates frequently demonstrated successful shifts. Student #19, initially wrote *“I don’t know,”* but later wrote *“No, turtles can have their sex determined by temperature and other species have different sex determination.”* And, some students who were already familiar with a range of sex determination mechanisms, like student #15, became more confident between their pre- and post-responses, shifting from *“Not sure. I know temperature dependence is a form of sex determination, like in alligators, but I feel as if there is chemical sex determination. I just can’t remember any examples,”* to *“No, it varies drastically over species, and vertebrates as a whole. Insects, fish, and reptiles all have drastically different sex determination strategies than plants or other invertebrates might have.”* Collectively, these students gained confidence and integrated more detailed examples into their answers.

While no students shifted towards less complexity in their knowledge, not all students completely integrated the complex course knowledge in their responses. Student #77 initially wrote *“Yes, always egg and sperm*” regarding if all sexually reproducing organisms use the same sex determination mechanism as in vertebrates, and then later wrote *“Yes—all sexes are based on gamete size. All organisms either produce large or small gametes.”* This answer is technically correct for gonochorists (organisms that only produce one type of gamete), but it does not take into account that hermaphrodites produce both big and little gametes at sometime in their lifetime.

Finally, some students failed integrate novel course content even if it was referenced in more than one way and on multiple days. For example, in response to the question about if sex is fixed, student #19 first wrote: *“A) I don’t think so. B) maybe*,” and then, in the post-assessment, wrote *“A) It can change in a lab with injections of hormones and surgery. B) I don’t think it can happen naturally*.” Although a lecture (on one day) and an in-class worksheet (on a different day) presented this material, this student ultimately did not demonstrate understanding of how certain fish species can change sex as adults, and the mechanisms by which they do so. Therefore, overall the way students integrated knowledge pieces from the course into their understanding of these topics varied.

#### Theme 2: Sex is at least a little bit complex, even in humans

Interestingly, the question about the complexity of humans, “*Does biology support or refute the idea that sex in humans is a binary trait controlled exclusively by simple genetics (i.e., it can be determined by a Punnett Square) that can be easily determined by external genitalia at birth*,” did not fit within the pattern described in Theme 1. Additionally, it generated a uniform pre-to-post change among students. Answers in the pre-assessment varied from supporting to refuting the statement. In contrast, in the post-assessment all students correctly refuted this statement. This change between the pre- and post-tests demonstrates that students applied some new pieces of knowledge in ways consistent with the course objectives by the end of the course and no students stopped applying relevant knowledge pieces. The multiple specific components of this question may have helped students to think more about this question than other societally entrenched topics.

Analysis of the post-assessment reveals that students differed markedly in their ability to cite relevant biological evidence when refuting the statement. In some cases, students clearly broadened and deepened the type of explanations they were able to give. For example, student #15 wrote in their pre-assessment: *“The XX genotype and the XY genotype combine to give 50% chance of XX (female) or 50% chance of XY (male). The external genitalia confirms the sex at birth.”* In their post-assessment, this student changed the knowledge pieces they used, drawing from presumably new knowledge: *“Sex is more on a spectrum due to circumstances where sex can’t fully be determined at birth. Biology refutes this [the idea in the question] due to the idea of intersex being applicable.”* Student #37 similarly changed the knowledge they referenced, initially writing *“Biology supports the idea that sex in humans is a binary trait, female has XX and male has XY, even when there’s extra sex chromosome humans are still determined as male or female,”* then shifting to, *“It’s not controlled exclusively by simple genetics. Because there are all kinds of other factors effecting sex in humans, such as hormones.”* Thus, these students articulated a more accurate and nuanced explanation of sex in humans in their post-assessments.

In other cases, students refuted the statement in both assessments, but adjusted their explanations, using reasonable arguments each time; this makes it harder to interpret how the course material impacted them. Student #77 initially wrote, *“Refutes: many people are intersex—i.e., born with different genetics for different outcomes. One example is a female presenting individual with a non-functioning y chromosome. Genetically XY, but phenotypically female*.” In their post-assessment they were more succinct, *“Refutes—biological sex exists as a spectrum.”* In the pre-assessment this student appeared to reference knowledge pieces that were already aligned with what was taught in the class, hence we cannot know if and how their knowledge was refined or expanded.

In one case, the student demonstrated that they knew the statement should be refuted in both assessments, but their post-assessment rationale was weaker than their pre-assessment rationale. Student #32 initially wrote, *“Biology could either support or refute this because a human could be born with external genitalia as one thing but have different hormones or reproductive organs internally.”* However, in their post-assessment they wrote, *“Biology refutes this idea because of our chromosomes. Maternally based, you can get the chromosome from either mom or dad and it is random but will be determined by external genitalia.”* For this student, it appears that the additional complexity that they learned in the course may have created confusion in the knowledge pieces they were drawing from, rather than clarity. We interpret this example as demonstrating transitional understanding, in which the student correctly understands that the biological evidence refutes the statement, yet they have not effectively integrated their new knowledge to the point where they could clearly express a coherent explanation for their answer.

In sum, all students who completed the post-test demonstrated an understanding that sex in humans has complexity, and all manifested some general knowledge on the topic. However, only a subset of students successfully demonstrated a clear, consistent integration of new knowledge to the level that they could distinguish nuanced vocabulary terms and succinctly express reasons or provide examples that support their general knowledge of the topic.

#### Pre-post assessment summary

In general, we found that students’ post-assessment responses were more closely aligned with the biology of sex content for topics that were less familiar (subtheme 1b) than for those that were more familiar and societally charged (subtheme 1a). This difference is not explained by differences in complexity; if anything, we expect the less familiar topics to be more complex. This finding may indicate that students found it easier to learn novel topics than to revise or adjust their deeply ingrained pre-existing knowledge about biology of sex. In the case of novel topics, students did not have to first identify inconsistencies between their pre-existing knowledge pieces and newly gained conflicting information and then refine their overall perspective. The choice to move away from deeply entrenched knowledge pieces likely requires reflection (i.e., conscious labor), practice, and a preponderance of convincing evidence. Thus, the pre-post assessments indicate that although our curricular intervention around biology of sex topics provided considerably more depth, detail, mechanisms, and emphasis on biodiversity, the student learning gains were more successful for novel topics than for topics associated with entrenched knowledge pieces.

The one exception to this categorization was the question analyzed in Theme 2, specifically regarding whether human sex is a simple binary. The specificity of this question and limitations put on the statement may have triggered students to be wary of the statement and helped them to critically analyze it, in comparison to more general questions like what sexes they are familiar with. The use of refute/support structured questions is established in the education literature (Erduran et al. 2004; Berland and Hammer 2012; Cavagnetto and Kurtz 2016; Allchin and Zemplén 2020), and the differences in how students responded to the question with this structure may demonstrate the fruitfulness of this question structure type. The ability of students to articulate a refutation also has practical applications, since it is the type of statement students may encounter discussions of in their everyday lives.

### Content relevance: Student perceptions of learning about biology of sex topics

To understand how students responded to our revised curriculum, we asked them to complete an ungraded reflection survey following the unit. Our goal in this reflection was to understand students’ perceptions of the knowledge taught in the course, and to gather further information about how students were integrating the knowledge pieces taught. Were students finding the complexity of integrating knowledge pieces difficult because they thought they were not important or rejected them, or were the challenges students experienced related to other reasons? Here we analyze student responses to the survey questions: *“Do you feel that learning about sex vs gender, sex determination, and sexual systems is important and relevant for students majoring in biology or similar majors? Why or why not?”* From our analyses we developed two themes: (1) biology of sex content is vital for a comprehensive education in biology, and (2) biology of sex content is culturally important. Many students’ responses spanned both themes.

#### Theme 1: Biology of sex content is vital for a comprehensive education in biology

Many students stated that biology of sex topics are a vital component of a modern, comprehensive biology education. Within this theme we identified two sub-themes: (a) biology of sex topics comprise essential basic knowledge, and (b) biological diversity is interesting and important. Many responses that fit within this theme were characterized by both sub-themes.

Students identified biology of sex topics as fundamental components of biology, such as student #19, “*Yes, because if a student is majoring in biology they should learn all aspects of biology. These are just more terms and ideas that someone going into science should know, especially if they are going into a career that will require them to know these terms very well*.” This student's response highlights both the foundational nature of the matierial and its practical relevance for future careers.

Other students, like #67, articulated an appreciation that scientific knowledge grows and changes over time; thus, learning biology of sex topics provides a way to engage with the most up-to-date scientific findings:

*I think it is important for biology students to study this because it affects how you interact with others and explore science. Many of the topics that we cover in classes are usually well-defined and contain information, and are slowly changing. Whereas this topic is less defined and is changing as more research is being done. So it allows students to keep an open mind and become more open to change*.

Students who discussed the importance of diversity within these topics often did so through expanding upon the topics as fundamental, such as student #98, *“I definitely think it is important because a lot of people tend to fall under the belief that sex = gender, and that is biologically not the case. There are numerous sexual systems on this earth, all that are equally successful in their own ways and it’s important to understand them all.”* Students in this sub-theme either called out the general importance of diversity specifically, or gave specific examples of diversity:

*I do think it’s important …. Because these topics are about things that occur naturally in the environment. Different organisms have different ways of sex determination, it could be genetic or it could be environmental; sexual systems also varies between species. So I do think it’s important to have an idea of what these topics are especially if majoring in biology related fields, because it’ll likely be helpful for the future*.

In the above comment, student #37 names examples that are relevant in understanding the developmental biology of variation across different types of organisms.

Two students still generally thought the material was important, but had more qualified answers. Student #56 discussed how the importance of this information was related to one’s career trajectory, with the material more or less relevant for different students:

*I feel like it could go both ways. It is important because if the students majoring in biology or similar majors is going into the research area it would be nice to know about sex determination and sexual systems. It might not be important if you aren’t going to be doing any experiments that will require that knowledge*.

Student #36 had different qualifications, noting that the information about sex was important, but that they thought gender (which was only discussed for about two minutes, in the context of differentiating sex and gender) was less relevant.

*I thought that some of this material was interesting, and probably important to learn about. I thought it was interesting to learn how different organisms can change their sex post maturation, and that some organisms' sex can be altered by chemicals or temperature. I didn’t find the gender portion to be as important towards biology or similar majors though. To me, gender seemed more about how someone feels, rather than their biology. I thought it was much more interesting to learn about how individuals can be intersex based off of chromosomal composition, or hormonal expression*.

Overall, students articulated robust support for the premise that biology of sex topics are important foundational concepts that interest them and support their professional goals as scientists. While one student noted that they thought that the gender content was less relevant, others, particularly in the quotes discussed in the next theme, explicitly stated that the inclusion of the gender content was important for student learning.

#### Theme 2: Biology of sex content is culturally important

Students also named the current cultural importance of understanding biology of sex topics in their response. Some students simply mentioned cultural contexts, often naming the importance of differentiating sex and gender, such as student #38: *“Yes, especially considering the current social and political climate. I think it is important that people understand that sex and gender are two separate categories.”* Other students provided more specifics, like student #55:

*I feel that learning all of these concepts is important and relevant for students majoring in biology or similar majors, because it helps to bring a whole understanding. Given the current political climate around gender topics, and the overwhelming amount of evidence to show dynamic sexual systems that do not adhere to a binary structure, I think that learning these topics helped me to understand the science behind our perceptions and the systems at work in nature*.

Some students, such as student #71 explicitly called out the importance of biological knowledge to understand humans with diverse gender identities:

*Yes, as it is a very “hot” or controversial topic, and as scientists we need to see information about these topics to better understand them for ourselves. I think this allows us to better understand people who feel these ways, (that [there] are not the two generic male and female sexes/gender’s) and the hardships they endure due to hate*.

Therefore, students underscored the connection with culture in a range of ways, with some providing a general reference to current social and political climates and others providing specifics, including how these topics are relevant for understanding how sex, gender, and reproduction are not binaries, or how binary-centric assumptions are associated with hate towards and hardships for people who live beyond these binaries.

Overall, through their reflection responses students vigorously affirmed that learning about the biology of sex and its diverse manifestations was interesting, foundational, and relevant for their education as biology majors and members of society. More specifically, students generally affirmed the premise that science involves refining and reorganizing ideas, that different contexts can help scientists appreciate new aspects, and that scientists and others should be open to the idea of experimentation, discussion, and reflection. Some students wrestled with the concept that scientific knowledge has to be interpreted and that scientific conclusions are shaped by human reasoning, cultural assumptions, and values. These responses demonstrate the need for critical reflection and conceptual flexibility. While one student noted that gender seemed less relevant in biology, many others articulated the need to discuss the inter-relationship of sex and gender to help them navigate current socio-political contexts.

In the current political climate, students’ perceptions of what belongs in a biology course may be of heightened concern to biology instructors, particularly in social climates where instructors are concerned about legislation or institutional norms that could limit how they teach about sex-related topics (Beatty et al. 2023; Choi et al. 2025). Understanding students’ perceptions of what belongs in a biology course—and why—helps instructors design curriculum and confidently approach potentially controversial subjects. Our findings demonstrate that students overall did believe this content belonged. Furthermore, students justified its inclusion by pointing to the topic's growing relevance in socio-political discussions. While collected in 2023 under a less heightened political atmosphere, our data show that political polarization does not deter students from engaging with sex-related content. Instead, societal division can actively fuel their demand for knowledge.

### Collective synthesis

Teaching challenging and complex content well requires a multiplicity of factors: effective teaching strategies that accommodate learner needs, instructors willing to find and apply those strategies, and learners that are willing to engage. One goal of this study was to assess how receptive students were to learning about biology of sex topics. Overall, we found that students expressed a strong desire to learn this material because they recognized it as foundational science and vital to a comprehensive understanding of biology, felt it could be useful to their future careers, and enjoyed and valued biodiversity. In addition, they recognized that cultural perceptions around sex are evolving, desired to be conversant with the biological basis for concepts relating to sex, and felt that biology of sex topics are relevant for a more inclusive society. Instructors wrestling with the social controversy of biology of sex topics can take heart from this data, which suggests that students are strongly motivated to engage with the material. Our findings build on prior research with similar findings (Casper et al. 2022; Eddy et al. 2026a) through providing data from all of the students in a single course with strategically designed content.

Finding effective strategies to teach the complexity of biology in ways that are simplified enough for students to understand while assisting them to grapple with nuanced definitions, detailed mechanisms, and organisms’ multiple ways of enacting biological goals is challenging. This task is even more challenging for biology of sex topics, where oversimplified and overgeneralized instruction and textbooks generate “common knowledge” blended with and enforced by societal narratives (Bazzul and Sykes 2011; Fuselier et al. 2018; Spaulding and Fuselier 2023; Jackson et al. 2024). The idea that sex is a discrete and universal binary is one such example of a commonly held but incorrect concept. As our findings demonstrate, upper-level biology courses, such as developmental biology, encounter this challenge when students confront detailed content that is incongruent with the simplified knowledge they may have referenced for years.

Our research demonstrates that attempting to teach the complexity of biology of sex topics simply by expanding the unit to include more depth, detail, mechanisms, and emphasis on biodiversity is not sufficient to support all students in learning this complexity. Our analysis of a course exam and pre-post assessments indicate mixed learning. Therefore, calls like those of Zemenick et al. (2022) and Casper et al. (2025) to center teaching biological complexity in teaching need to be complemented by classroom-tested pedagogical strategies to effectively teach this complexity. Students particularly struggled to integrate the course content into their existing knowledge when they needed to navigate deeply rooted knowledge pieces that were not congruent with the course material. Thus, we provide some of the first evidence to demonstrate the challenges in transforming how biology students conceptualize biology of sex topics. Further research is needed to identify specific interventions and strategies that help students effectively engage with the complexity of biology of sex topics.

#### Future research: Pathway towards systemic change

Systemic change in how we teach biology of sex topics is needed to better support student learning about the complexity of these topics. Our findings point to this need for change, but do not provide direct guidance for this change. However, others’ research on parallel topics do provide guidance. Strategies that have been effective for teaching potentially socially contentious topics, such as evolution, may be particularly beneficial, and future research applying the strategies described in this section, and potentially others, is needed to improve teaching about biology of sex topics.

Researchers studying a range of topics, from evolution to photosynthesis, found cognitive bridging and identity threat protection strategies to be pedagogically successful (diSessa 2008; Barnes and Brownell 2017; Kahan 2017; Vidak et al. 2019; Aleknavičiūtė et al. 2023; Aini et al. 2024, 2025; Pacaci et al. 2024). Cognitive bridging involves connecting new content to students’ existing knowledge pieces (Vidak et al. 2019) and identity threat protection strategies help avoid putting students in the position of feeling like the course content is in conflict with their beliefs. While there is not yet research applying these strategies to biology of sex topics, Eddy et al. (2026b) describe the implementation of cognitive bridging and identity threat protection for biology of sex topics in a range of high school or undergraduate contexts and their supplement includes curricular materials.

Along with these strategies, which have been fruitful in topics with parallel challenges, naming the complexity of biology of sex topics, highlighting examples of common oversimplifications and overgeneralizations, discussing the biases we bring to sex-related topics through our lens of being mammals and humans, and carefully integrating accurate language use about sex-related topics may help student learning (Casper 2026; Casper and Warkentin 2026; Eddy et al. 2026b; Warkentin et al. 2026). Creating clarity about what we mean by the term “sex” in a specific context, rather than arguing for a single right definition of sex, and structuring complexity around common simplifications, can create space for differing concepts to co-exist around sex-related topics—much as they do for species-related concepts (Hey et al. 2003; Haveman 2013; Sandberg et al. 2025; Eppley et al. 2026; Warkentin et al. 2026).

One exciting and potentially relevant paradigm for teaching complexity is Dauer and Dauer’s (2016) complexity theory, which emphasizes that biological systems are inherently complex and that students must develop reasoning skills to navigate this complexity. As students learn, they realize that their initial explanations are inadequate and oversimplified. For biology of sex topics, providing concrete examples that connect topics such as genetics, environment, anatomy, hormones, and evolution, drawing from students’ existing knowledge, and scaffolding common oversimplifications into a larger framework of complexity may help students integrate this broader complexity into their pre-existing knowledge. This interconnectivity may decrease frustrations and rejections of new knowledge pieces related to conflicts with pre-existing knowledge pieces. As Casper (2026) discusses, this approach creates a scaffolding for contextualized simplified content—teaching simplifications may be necessary to support student learning, however providing the larger context of biological variation and complexity can help students situate these simplifications within biological complexity. For example, instructors often initially teach physics from a friction-free perspective, but our lived experience helps us avoid inappropriately applying this oversimplification to daily life. Likewise, in biology our students need structured scaffolding to understand that much of what they initially learn in classes is the biological equivalent of frictionless physics.

Teaching sex-related topics from a theory-informed perspective that engages with the knowledge pieces students enter the classroom with is particularly important in current contexts. The value of using identity-threat protection strategies and cognitive bridging techniques to teach other polarized topics indicates that these strategies may be fruitful for sex-related topics. Approaching these strategies from a KiP perspective, considering the range of pieces of knowledge students are grappling with and using other research-based pedagogical strategies that explicitly target the challenges of teaching biology of sex topics may provide pathways for instructors to provide context and structure to topics.

#### Limitations

Our findings are from a small study and have several limitations: (a) attendance was not taken in class; hence, it is unknown which students attended the class periods that aligned with course materials covered in the pre-post assessment; (b) while we emphasized a non-binary, biodiverse perspective in the biology of sex unit, other units of the course that touched on mammals adhered to a mammal/human-centric binary sex perspective without particularly highlighting non-binary examples; and (c) only ~20% of enrolled students completed both pre- and post-assessments. Additionally, student answers were brief, and we do not have information about how certain students were of their answers, which could help us understand students’ prior knowledge. From a learning perspective, because only one unit was changed, the potential mixed messages in the course may have reinforced overgeneralizing simplifications across taxa, and limited students’ shifts in the knowledge pieces referenced in the end-of-course post-assessment. Furthermore, the order that material was taught in may have influenced the exam results. The sex-related content was the earliest-taught material on exam three; it was taught in weeks four and five, the other content was taught in weeks five and six, and the exam was given in week seven. Lastly, as with all human subjects research, our data are limited by the often non-random way students made consent decisions. We cannot know if the responses from the students who did not consent to participate would differ from those who did consent. We also cannot know how students’ responses would differ in the socio-political climate at the time of final manuscript drafting, which differs from the climate in which we performed this study.

## Conclusion

Effectively teaching the complexity of sex-related topics is important for a range of reasons, including to support biological knowledge that is foundational for future researchers, the belonging of students across a range of identities, and to avoid unintentionally creating environments where oversimplifications and overgeneralizations of biology can be used to support discriminatory laws (Long et al. 2021; Zemenick et al. 2022; Casper, Fuselier, et al. 2025; Eddy et al. 2026a).

Our findings show that teaching the complexity and variation of sex-related topics is not enough by itself to support student learning. Even in an upper-level undergraduate course students had oversimplified foundational knowledge that could limit their effectiveness in integrating new, more complex knowledge into their understanding of the world. Through the insights of KiP, regarding the different knowledge pieces students drew from across topics with a range of prior familiarity, we have identified some of the obstacles which limit the success of typical teaching strategies. On a positive note, students appear strongly receptive to the rationale for learning about biology of sex topics. Taken together, these findings support the need for development of specific strategies and interventions to improve effective learning for biology of sex topics. Educational theories that focus directly on how to support learning complex material have the best potential for providing a path forward (Bender et al. 2012).

## Supplementary Material

icag149_Supplemental_File

## Data Availability

The participants of this study did not give written consent for their data to be shared publicly, so due to the sensitive nature of the research supporting data are not available.

## Notes

From the symposium “Sex across origins: Questioning animal-centric assumptions and developing integrative frameworks” presented at the annual meeting of the Society for Integrative and Comparative Biology, January 3–7th, 2026.
