# Supplementary material for: Centering Biodiversity: Integrating the Complexity of Sex Into a Developmental Biology Course
Source: Integr Comp Biol. 2026 Aug 10;66:icag149. doi: 10.1093/icb/icag149 (PMC13560086; doi:10.1093/icb/icag149)
Supplement: Supplementary file 1 [file icag149_supplemental_file.pdf]

Supplement 1: Curriculum on the Biology of Sex: Determination, Development, and Diversity

Learning objectives:

- Explain how scientific frameworks and explanatory models shape biological understanding of sex and sex determination, including how meaning is constructed within biological sciences.
- Describe how biological sex emerges from multilevel, interacting processes, including chromosomal, genetic, hormonal, and environmental influences.
- Analyze genotype–phenotype relationships to explain how diverse male- and female-typical human phenotypes arise from chromosomal configurations.
- Evaluate why biological sex does not function as a strictly binary trait, using comparative and developmental biological evidence.
- Compare sex determination mechanisms across taxa, including mammals, invertebrates (e.g., *Drosophila*), fishes, and other clades, highlighting shared principles and key differences.
- Explain mammalian sex determination as a gene regulatory and developmental process, including the role of the bipotential gonad, the *SRY* gene, hormonal signaling, and primary and secondary sex determination.
- Interpret biological variation in sex development, including variations in sex characteristics (VSCs/DSDs), as evidence that sex determination operates as a complex, multilevel biological process.
- Apply biological terminology accurately and respectfully when discussing sex and variation in living organisms.

Content:

Meaning-making in biological science: How scientific frameworks and explanatory models shape understanding of biological sex and sex determination.

> **Epistemological perspectives:** Ways of knowing in biology that influence how sexes are defined, studied, and interpreted.
>
> **Polysemy of "sex":** The multiple biological meanings of the term *sex* across genetic, developmental, anatomical, and reproductive contexts.
>
> **Multilevel biological processes:** How multiple interacting biological processes contribute to the development of an individual's sex.
>
> **Genotype–phenotype relationships:** How diverse human male- and female-typical phenotypes arise relative to chromosomal configurations.
>
> **Beyond a simple binary:** In what biological sense sex does not operate as a strictly binary trait.
>
> **Comparative mechanisms of sex generation:** Which biological processes generate sexes within a species, given that no single defining feature applies universally.
>
> **Sex versus gender:** How biological sex, as an attribute of an individual organism, differs conceptually and biologically from gender.

Sex determination in mammals: An overview of the biological mechanisms that establish sex during mammalian development.

Chromosomal sex determination systems: How XX and XY chromosomal configurations function as the primary sex determination system in mammals.
The testis-determining factor: The role of the *SRY* gene and the experimental evidence supporting its involvement in initiating testis development.
Gene regulatory networks: Recognition that *SRY* functions within a broader, tightly regulated network of genes rather than acting as a sole determinant of sex.
Primary and secondary sex determination: How sex-related traits are established through sequential processes that determine gonadal fate and subsequent sexual differentiation.
Hormonal interpretation of genotype: How XX and XY genotypes are translated into sex-specific phenotypes through hormonally mediated developmental pathways.
Developmental models of sex determination: Conceptual models of mammalian sex determination beginning with the bipotential gonad.
Biological variation: Variations in sex characteristics (VSCs), also referred to clinically as differences of sex development (DSDs), and their relevance to understanding sex as a multilevel biological process.

Sex determination in invertebrates: An overview of the diverse genetic and developmental mechanisms by which sex is determined in invertebrate taxa.

Chromosomal organization and sexual phenotypes in *Drosophila*: How *Drosophila* sex chromosomes contribute to sex-specific developmental outcomes and phenotypic traits.
Gynandromorphs: Cases in which sex is not determined or expressed uniformly across an entire organism, illustrating cell-autonomous sex determination.
Genetic basis of sexual dimorphism: Examples of *Drosophila* mutant phenotypes that alter sexually dimorphic traits and reveal underlying genetic control mechanisms.
***Drosophila* sex determination pathway:** The genetic interpretation of the X-to-autosome ratio and the central regulatory role of the *Sex-lethal (Sxl)* gene in directing sex-specific development.

Factors Influencing the Phenotypic Expression of Chromosomal Sex

Determinants of phenotypic sex expression: An overview of genetic, molecular, and environmental factors that influence how chromosomal sex is translated into observable traits.

Dosage compensation: Mechanisms that balance gene expression between individuals with differing numbers of sex chromosomes.

X-chromosome inactivation: Molecular processes that regulate X-linked gene expression and their consequences for phenotypic variation.

Environmental sex determination: The role of environmental cues, including temperature and social context, and their interaction with hormonal signaling.

Sex determination in fish: Mechanisms underlying genetic and environmental sex determination in fishes, including the biological basis for sex change in some species.

Sexual Systems: Diversity, Plasticity, and Evolution

Sexual systems: An overview of the range of organizational patterns by which sexes are distributed among individuals within species.

Comparative sexual systems: Comparison and contrast of major sexual systems, including androdioecy, hermaphroditism, trioecy, gynodioecy, and gonochorism.

Life-history diversity: Variation in life-history strategies related to sex and reproduction, including sequential hermaphroditism.

Phenotypic plasticity: The role of environmental and developmental influences in shaping sex expression and reproductive function.

Evolution of sexual systems: The ecological, genetic, and evolutionary processes that give rise to and maintain diverse sexual systems.

Variation in sex determination mechanisms: How methods of sex determination differ widely across taxonomic clades.

Evolution of sex determination pathways: How sex determination systems and sex chromosomes can originate, diverge, and evolve over evolutionary time.
